# Determination of Haloacetic Acids in Bottled and Tap Water Sources by Dispersive Liquid-Liquid Microextraction and GC-MS Analysis

**DOI:** 10.1155/2014/695049

**Published:** 2014-09-11

**Authors:** Mohsen A. Al-shatri, Abdulmumin A. Nuhu, Chanbasha Basheer

**Affiliations:** ^1^Department of Chemistry, King Fahd University of Petroleum and Minerals, KFUPM Box 1059, Dhahran 31261, Saudi Arabia; ^2^Department of Chemistry, Ahmadu Bello University, PMB 1069, Zaria, Kaduna 2222, Nigeria

## Abstract

Haloacetic acids are toxic organic pollutants that can be formed as by-products of disinfection of water by chlorination. In this study, we developed a fast and efficient method for the determination of six species of these compounds in water using dispersive liquid-liquid microextraction followed by GC-MS analysis. To be suitable for GC analysis, the acidic analytes were derivatized using *n*-octanol. One-factor-at-a-time optimization was carried out on several factors including temperature, extraction time, amount of catalyst, and dispersive solvent. The optimized conditions were then used to determine calibration parameters. Linearity, as demonstrated by coefficient of determination, ranged between 0.9900 and 0.9966 for the concentration range of 0.05–0.57 *µ*g/L. The proposed method has good repeatability; intraday precision was calculated as %RSD of 2.38–9.34%, while interday precision was 4.69–8.06%. The method was applied to real samples in bottled water and tap water sources. Results indicated that the total concentrations of the analytes in these sources (2.97–5.30 *µ*g/L) were far below the maximum contaminant levels set by both the World Health Organization and the United States Environmental Protection Agency. The proposed method compared favorably with methods reported in the literature.

## 1. Introduction

When chloride or bromide is oxidized in water, some of the oxidized species can react with natural organic matter (NOM) to form disinfection by-products (DBPs) called haloacetic acids (HAAs). These HAAs are found in industrial wastes and as by-products of water chlorination [1, 2]. They are also found in other materials such as drugs and dyes [3]. They are highly soluble in water and toxic to humans [4], animals [5], and plants and algae [6].

Toxicological studies have shown that some HAAs (especially trichloroacetic acid and dichloroacetic acid) and other DBPs can cause adverse effects, including cancer, in laboratory animals [7, 8]. Because of this serious health risk, regulatory action has been taken to control the levels of these DBPs in drinking water. While the World Health Organization (WHO) has stipulated the maximum contaminant levels (MCL) for monochloroacetic acid, dichloroacetic acid, and trichloroacetic as 20, 50, and 200 *μ*g/L, respectively [8], the sum of concentrations of HAAs should not be greater than 60 *μ*g/L according to the United States Environmental Protection Agency (USEPA) [9].

Various methods have been reported for the determination of HAAs in different matrices. Liquid chromatographic methods [10–14] and capillary zone electrophoresis [14] have been employed in this regard. These methods did not involve any derivatization steps. However, gas chromatographic (GC) methods are the most widely utilized methods due to their inherent advantages of high resolution, rapid separation, low cost, and easy coupling with sensitive and selective detectors, such as electron capture (ECD) and mass spectrometric (MS) detectors [15–18]. Due to the high polarity and low volatility of HAAs, a prior derivatization step is necessary before GC determination [19]. This derivatization can be performed simultaneously with, or preceded by, an extraction step. The extraction step can be achieved by liquid-liquid extraction (LLE) [20], solid-phase extraction (SPE), and supercritical fluid extraction (SFE) [21]. Microextraction techniques, such as solid-phase microextraction (SPME) and single-drop microextraction (SDME), have been harnessed for the quantification of HAAs in water using GC-MS [22–27]. A new microextraction technique called the dispersive liquid-liquid microextraction (DLLME) has been described [28].

In the postextraction derivatization, the derivatization of HAAs to short-chain esters using different reagents, such as diazomethane [13], acid-alcohol [29–31], dimethyl sulphate [16], or BF3-methanol [32], is performed. Pentafluorobenzyl bromide (PFBBr) was used as a new and suitable reagent for the derivatization of various HAAs [33–35].

In the second approach, derivatization of HAAs is performed simultaneously with extraction in solvent microdrop by using* n*-octanol as extractant solvent and derivatization reagent [36]. However, this approach is very tedious and applicable only for small volume of clean samples. In addition, the approach is not suitable for environmental samples which most often contain interfering substances.

Thus, our objective in this current work was to develop a sensitive and simple dispersive liquid-liquid microextraction (DLLME)/GC-MS method for the determination of six haloacetic acids in water matrix of several bottled and tap water sources.

## 2. Materials and Methods

### 2.1. Reagents and Chemicals

HPLC-grade solvents were purchased from J. T. Baker (NJ, USA). Analytical grade standard mixture of six haloacetic acids containing monochloroacetic acid (MCAA), dichloroacetic acid (DCAA), trichloroacetic acid (TCAA), monobromoacetic acid (MBAA), dibromoacetic acid (DBAA), and bromochloroacetic acid (BCAA) (2000 *μ*g/mL each in methyl tert-butyl ether (MTBE)) was purchased from Supelco (Supelco Park, PA, USA). Ultrapure water was obtained using a Milli-Q-water purification system (Millipore, Bedford, MA, USA).

### 2.2. Preparation of Working Standard

The working standard solution of six HAAs was prepared at a concentration of 80 ppm by diluting the stock standard solution at 1 : 25 with acetone. The standard solution was stored at 4°C and warmed to ambient temperature before use.

### 2.3. Sampling Locations and Sample Collection

Four tap water samples and three bottled water samples were obtained within the Eastern Province of Saudi Arabia. The bottled water samples, NOVA, NEST, and PURE were purchased from a local market in Al-Khobar. Tap water samples were collected from different locations; KS was collected from storage tank beside Carrefour Mall in Al-Khobar, PS23 was from water filling station at Street 23 in Al-Thoqba, and PS and B822 were obtained from water treatment station and Building 822, respectively, both at King Fahd University of Petroleum and Minerals. The chosen taps were the most frequently used. Any external fittings such as filters were first removed and any possible contaminant around the spout was wiped off with a clean cloth before collecting the samples. The water samples were collected in 1 L screw cap plastic bottles with no headspace after flushing. The samples were collected with as little agitation or disturbance as possible. These samples were returned to the laboratory where analysis was preformed immediately.

### 2.4. Instrumentation

The six HAAs were separated and detected using gas chromatography-mass spectrometry (Agilent, 6890 GC-MS) system. Chromatographic separation was accomplished on HP-5MS 5% phenyl methyl siloxane (30 m × 0.25 mm × 0.25 mm nominal) from J&W Scientific. The column was initially maintained at 40°C for 1 min and then increased to 180°C at the rate of 25°C/min. This was held for 11 min followed by another ramping to 250°C at 30°C/min. This was maintained for 2 min. The GC-MS interface and the ion source temperatures were set at 200°C. Helium (99.999%) at a head pressure of 50 kPa was used as carrier gas with flow rate of 2.0 mL/min. The split/splitless injector temperature was set at 250°C. The injection volume was 0.2 *μ*L and injection was performed using a 10 *μ*L GC microsyringe.

### 2.5. Extraction and Derivatization of HAAs

Many studies have been carried out to investigate the effect of acidity on the determination of HAAs. In one of these studies, Saraji and Bidgoli [36] have tested the effect of different amounts of concentrated sulphuric acid (1, 3, 5, 7, 10% v/v) on extraction efficiency and found that the highest response was obtained with 10% H_2_SO_4_. This amount was, therefore, adopted in our study.

Solvent used for extraction in GC-related methods must have good affinity for target compound, low solubility in water, enough stability over the extraction time, and an excellent gas chromatographic behavior [36]. In the present study we used* n*-octanol because it has lower density than water, making it easier to obtain the organic solvent phase after extraction and it readily mixes with the dispersive solvent used.

Increase in solution ionic strength has been found to improve extraction efficiency. Sodium chloride, NaCl, is generally used in this regard. However, in the extraction of HAAs from water samples, the use of NaCl is discouraged [9, 13, 32] because it may contain trace levels of bromide which has been shown to promote the formation of brominated HAAs. As a substitute for this, Saraji and Bidgoli [36] have studied the effect of different amounts of Na_2_SO_4_ and found that 12.4% salt concentration produced the highest extraction efficiency. This amount was consequently utilized in our study.

Simultaneous derivatization and liquid-liquid microextraction (LLME) were carried out using* n*-octanol as extractant solvent and derivatization reagent. A 10 mL vial (Supelco, Bellefonte, PA, USA) containing 1 mL* n*-octanol and 5 *μ*L trifluoroacetic anhydride (TFAA) catalyst was spiked with standard mixture of HAAs and made to a volume of 7 mL with distilled water containing 10% (v/v) concentrated H_2_SO_4_ and 12.4% (w/v) Na_2_SO_4_ to give the analyte concentration of 0.5 *μ*g/L. The solution was thoroughly mixed in an ultrasonic water bath. Acetone (AC), acetonitrile (ACN), tetrahydrofuran (THF), ethanol (ET), and methanol (ME) were tested in order to find the best and most appropriate dispersant solvent for optimal performance. Other variables that affect derivatization reaction yield and extraction efficiency, such as temperature (25°C, 60°C, and 100°C), amount of TFAA (2 *μ*L, 5 *μ*L, 10 *μ*L, 15 *μ*L, 30 *μ*L, and 50 *μ*L), and sonication (extraction) time, were also optimized.

## 3. Results and Discussion

### 3.1. Effect of Temperature

**Figure 1:**
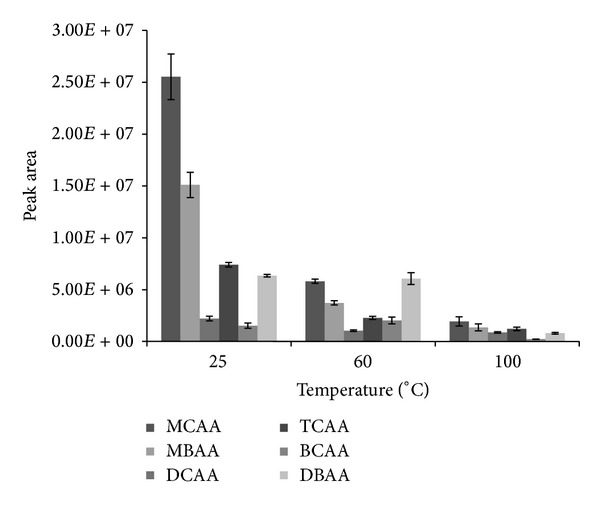
Effect of temperature on the simultaneous liquid-liquid microextraction and derivatization of 0.5 *μ*gL^−1^ haloacetic acids at experimental conditions of 5 min reaction time, 5 *μ*L TFAA as catalyst, and 1 mL *n*-octanol as both extractant solvent and derivatization reagent.

Increase in temperature means an increase in kinetic energy and thermodynamic efficiency of diffusion process during the extraction. This leads to reduction in the time required to reach equilibrium. On the other hand, a high temperature may lead to the loss of the solvent, thus decreasing the extraction yield [36]. In order to obtain the optimum temperature, we tested three different degrees (25, 60, and 100°C) under fixed conditions of 1 mL* n*-octanol, 5 *μ*L TFAA, and 5 min extraction time. As a result Figure 1 shows, at 100°C, that peak areas for all the analytes were between 2.20 × 10^5^ and 1.94 × 10^6^. At 60°C, this range was between 1.04 × 10^6^ and 6.06 × 10^6^; response for all analytes was slightly improved compared to that obtained at 100°C. This improvement was significant at 25°C where the range of the response in peak areas was between 1.53 × 10^6^ (BCAA) and 2.55 × 10^7^ (MCAA); only BCAA showed a weaker response at this temperature compared to 60°C. The low responses for the analytes at high temperatures of 60°C and 100°C might be due to the loss of the extraction solvent and the resultant decrease in extraction efficiency. In general, therefore, 25°C gave the best performance and was chosen as the optimized temperature in this study.

### 3.2. Effect of Reaction Time

**Figure 2:**
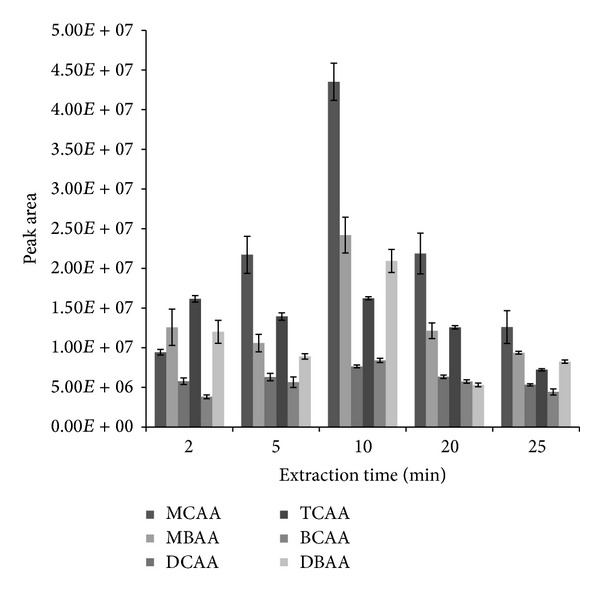
Effect of reaction time on the simultaneous liquid-liquid microextraction and derivatization of 0.5 *μ*gL^−1^ haloacetic acids at experimental conditions of 5 *μ*L TFAA as catalyst, temperature of 25°C, and 1 mL* n*-octanol as both extractant solvent and derivatization reagent.

In order to examine the effect of reaction time on the extraction and derivatization procedure, different times of 2, 5, 10, 20, and 25 min were tested at 25°C. Other fixed experimental conditions include 1 mL* n*-octanol and 5 *μ*L TFAA.  Figure 2 shows the result of this experiment. At 10 min, the best response obtained was for MCAA (peak area, 4.35 × 10^7^) while the worst response was for DCAA (peak area, 7.64 × 10^6^). However, the response for all the analytes showed improvement compared to other extraction times, except TCAA which had the same response of 1.62 × 10^7^ both at 2 min and at 10 min extraction times. The result indicates that 10 min achieved the highest extraction yield and was enough to bring the derivatization reaction to completion. Therefore, 10 min was adopted as the optimum extraction and derivatization time in this work.

### 3.3. Effect of Amount of Catalyst

**Figure 3:**
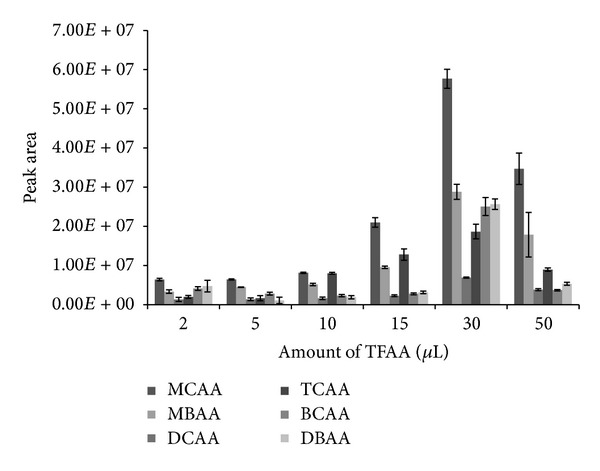
Effect of the amount of catalyst (TFAA) on the simultaneous liquid-liquid microextraction and derivatization of 0.5 *μ*gL^−1^ haloacetic acids at experimental conditions of 10 min reaction time, 25°C temperature, and 1 mL* n*-octanol as both extractant solvent and derivatization reagent.

The effect of the amount of TFAA on the derivatization reaction between haloacetic acid and* n*-octanol was investigated by testing different amounts of TFAA (2 *μ*L, 5 *μ*L, 10 *μ*L, 15 *μ*L, 30 *μ*L, and 50 *μ*L) at fixed experimental conditions of 25°C, 1 mL* n*-octanol, and 10 min reaction time.  Figure 3 shows the peak areas of the six HAAs compounds under study. From 10 to 30 *μ*L of the catalyst, there was consistent increase in the response of all the analytes as demonstrated by their enhanced peak areas. However, at 50 *μ*L, the response of the analytes depreciated. Probably at this volume, the analytes could not interact efficiently with the extraction solvent leading to the lower extraction efficiency as compared with 30 *μ*L volume. This result, therefore, clearly indicates the advantage of 30 *μ*L over other tested volumes of the catalyst. For this reason, we selected this volume in furtherance of the one-factor-at-a-time optimization process.

### 3.4. Effect of Dispersive Solvent

**Figure 4:**
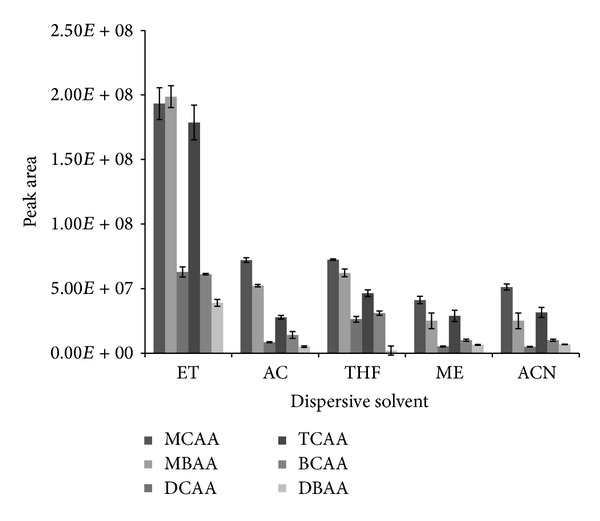
Effect of type of dispersive solvent on the simultaneous dispersive liquid-liquid microextraction and derivatization of 0.5 *μ*gL^−1^ haloacetic acids at experimental conditions of 10 min reaction time, 25°C temperature, 30 *μ*L TFAA as catalyst, and 1 mL* n*-octanol as both extractant solvent and derivatization reagent.

To examine the effect of dispersive solvent on derivatization reaction and simultaneous extraction of the different analytes, six different solvents were tested: ethanol (ETOH), methanol (MEOH), acetone (AC), acetonitrile (ACN), and tetrahydrofuran (THF). Experiment was performed at fixed conditions of 1 mL* n*-octanol, 25°C, and 30 *μ*L of TFAA for the duration of 10 min.  Figure 4 summarizes the result obtained for this experiment. It clearly shows that dispersion using ethanol has an added advantage over nondispersive approach as indicated by the appreciable increase in peak areas (3.90 × 10^7^ − 1.99 × 10^8^) of the analytes compared to the result obtained (6.96 × 10^6^ − 5.77 × 10^7^) in the preceding optimization step (Figure 3) that did not involve the use of any dispersive solvent. When compared to other types of dispersive solvents, response for all analytes, without exception, was highest when ethanol was used. Hence ethanol was selected as the dispersive solvent and the name dispersive liquid-liquid microextraction (DLLME) was adopted for the developed method.

From the results obtained, optimized conditions for this study were 1 mL* n*-octanol as extractant solvent and derivatization reagent, 1 mL ethanol as dispersive solvent, 30 *μ*L TFAA as catalyst, temperature of 25°C, and reaction time of 10 min.

### 3.5. Quality Assurance

The optimized conditions above were applied in measuring precision using both intraday and interday relative standard deviations (%RSD) and linearity as determined by spiking ultrapure water with standards of HAAs at concentrations ranging from 0.05 to 0.57 *μ*g/L. These results are presented in Table 1. The results indicate good linearity as demonstrated by the coefficients of determination (*r* ^2^) between 0.9900 and 0.9969. Intraday precision for the different analytes ranged from 2.38 to 9.34%, while interday precision was 4.69–8.06%, indicating good repeatability of the proposed method. The interday precision was determined through consecutive extractions for a period of five days. This and other parameters such as sample volume and derivatization time show that the proposed method compared favorably with what is reported in the literature (Table 2).

In order to test the applicability of this method to real samples, water samples from different sources were analyzed (Table 3). With the exceptions of KS and NOVA samples in which TCAA was not detected, all other samples contained all the species of HAAs determined. For the tap water sources, the highest total amount of HAAs (3.83 *μ*g/L) was recorded in B822 sample while the lowest (3.1 *μ*g/L) was recorded in KS because TCAA was not detected in this sample. For the bottled water samples, DBAA was found in significantly high amount in PURE. As a result, the highest total amount of HAAs (5.30 *μ*g/L) was obtained in this sample. However, the lowest value (2.97 *μ*g/L) was recorded in NOVA because TCAA was not detected in this sample. These results show that the values determined for MCAA, DCAA, and TCAA were far below the WHO maximum limits of 20, 50, and 200 *μ*g/L, respectively [8]. In addition, the total species of HAAs in each sample was below the 60 *μ*g/L maximum limit adopted by USEPA [9].

## 4. Conclusions

Dispersive liquid-liquid microextraction followed by GC-MS analysis was employed for the determination of six species of haloacetic acid in water matrix. This method utilized* n*-octanol as extractant solvent and reagent for the simultaneous derivatization of these polar analytes. The method was relatively fast as the simultaneous derivatization and extraction procedure was completed in 10 min. Both intraday and interday precision showed that the method had good repeatability. This and other parameters tested showed that the proposed method compared favorably with methods reported in the literature. The method was successfully applied to the quantification of the analytes in real samples of bottled and tap water sources. Concentrations determined indicated that these water sources contained HAAs at levels that were lower than the maximum contaminant levels prescribed by WHO and USEPA.

## Figures and Tables

**Table 1:** Calibration data and repeatability of the proposed method.

Analyte	Linear equation	*r* ^2^ ^a^	%RSD^b^	
			Intraday (*n* = 3)	Interday (*n* = 5)
MCAA	*y* = 8*E* + 06*x* − 4*E* + 06	0.9969	5.41	5.77
MBAA	*y* = 4*E* + 08*x* − 1*E* + 07	0.9900	9.34	7.02
DCAA	*y* = 1*E* + 08*x* − 5*E* + 06	0.9949	2.38	4.69
TCAA	*y* = 3*E* + 08*x* − 1*E* + 07	0.9956	9.88	8.06
BCAA	*y* = 1*E* + 08*x* − 4*E* + 06	0.9948	3.10	4.88
DBAA	*y* = 7*E* + 07*x* − 3*E* + 06	0.9951	6.96	5.22

^a^Coefficient of determination.

^b^Relative standard deviation.

**Table 2:** Comparison between proposed method and methods reported in the literature for HAAs determination in water.

Method	RSD (%)	D.R^a^	D.T^b^ (min)	Sample volume (mL)	Reference
Evaporate-SPME-GC-MS	6.3–7.9	Acidic ethanol	10	30	[16]
HS-SPME-GC-ECD	6.3–10.9	Dimethyl sulphate	5	10	[19]
LLE-GC-ECD EPA 552-3	0.36–4.0	Acidic methanol	120	40	[32]
LLE-GC-MS-MS	0.9–19.9	PFBBr	180	1	[35]
SDME-GC-MS	5.1–8.5	Octanol	20	3	[36]
LLE-GC-ECD EPA 552-1	7–59	Diazomethane	30	100	[37]
HS-HFLPME-GC-MS-*µ*ECD	5–12	Acidic methanol	NA	10	[38]
*µ*-SPE-UPLC-UV	0.03–7.40	—	—	20	[39]
LLE-ESI-MS	NA	NA	NA	188	[40]
UPLC-MS	NA	NA	NA	30	[41]
DLLME-GC-MS	2.38–9.34	Octanol	10	7	Proposed method

^a^Derivatization reagent.

^b^Derivatization time.

**Table 3:** Application of the proposed method on bottled water and tap water sources.

	Average concentrations (*µ*g/L)						
Analyte	Tap water sources			Bottled water sources			
	B822	PS	PS23	KS	NEST	NOVA	PURE
MCAA	0.69	0.66	0.70	0.63	0.72	0.71	0.75
MBAA	0.65	0.70	0.75	0.70	0.60	0.57	0.76
DCAA	0.64	0.54	0.56	0.54	0.53	0.55	0.65
TCAA	0.61	0.53	0.53	nd	0.58	nd	0.74
BCAA	0.62	0.50	0.52	0.61	0.58	0.55	0.82
DBAA	0.62	0.62	0.59	0.62	0.60	0.59	1.58

Nd: not detected.
